# A comparative study of single-stage bilateral vs. unilateral medial unicompartmental knee arthroplasty on complications, clinical outcomes, and costs

**DOI:** 10.3389/fsurg.2024.1470421

**Published:** 2024-10-02

**Authors:** Kao-Chang Tu, Han-Ting Shih, Shun-Ping Wang, Kun-Hui Chen

**Affiliations:** ^1^ Department of Orthopaedics, Taichung Veterans General Hospital, Taichung, Taiwan; ^2^ Doctoral Program in Tissue Engineering and Regenerative Medicine, National Chung Hsing University, Taichung, Taiwan; ^3^ Department of Post-Baccalaureate Medicine, College of Medicine, National Chung Hsing University, Tai-chung, Taiwan; ^4^ Department of Computer Science and Information Engineering, Providence University, Taichung, Taiwan

**Keywords:** unicompartmental knee arthroplasty, bilateral, unilateral, complications, clinical outcomes, costs

## Abstract

**Background:**

This retrospective study aimed to evaluate the short-term recovery and cost-effectiveness of bilateral unicompartmental knee arthroplasty (UKA) compared to staged unilateral UKA. The study analyzed postoperative pain scores, medical costs, and complications in patients with knee osteoarthritis who underwent these procedures.

**Methods:**

A total of 226 patients who received either unilateral UKA (Group A, *n* = 170) or bilateral UKA (Group B, *n* = 56) using the mobile-bearing UKA were included in the study. Patient demographics, surgical details, postoperative pain scores, knee range of motion, length of hospital stay, self-controlled analgesic use, total medical costs, and complications were retrospectively collected from medical records.

**Results:**

The demographic characteristics were comparable between the groups. Group B had a longer surgical time and higher medical costs than Group A. However, there were no significant differences in hospital stay, pain scores, or knee range of motion between the two groups. Complications were infrequent and not significantly different. Insert dislocation and loosening were the most common complications. Patient-controlled analgesia effectively reduced pain scores in Group A but not in Group B.

**Conclusion:**

Bilateral UKA does not significantly affect hospital stay, postoperative pain, or complications compared to unilateral UKA. Although bilateral UKA requires longer surgical time and incurs higher costs, it offers the potential benefit of reducing anesthesia-related complications and overall health insurance expenditures. This study recommends bilateral UKA as a suitable option for patients with bilateral knee osteoarthritis, given its comparable short-term outcomes and potential cost-saving advantages.

**Level of Evidence:**

III

## Introduction

The adoption of unilateral unicompartmental knee arthroplasty (UKA) has seen a significant increase in recent years. In terms of survival rates, medial UKA has demonstrated excellent outcomes in both short- and long-term follow-up studies. For example, Rudy Sangaletti's et al. research reported a 5-year survival rate of 100% (1), while Stefano Marco Paolo Rossi et al. documented a 15-year survival rate of 90% (2). Additionally, in Rudy Sangaletti's study, the 10-year survival rate for lateral UKA in younger patients was found to be 93% (3). This trend can be attributed to several key factors, particularly advancements in surgical techniques that have enabled more precise procedures. Additionally, patients benefit from reduced bone and soft tissue intervention, smaller incision wounds, faster post-operative recovery times, improved range of motion, and greater ease in addressing potential challenges that may arise during subsequent revision procedures. As a result, UKA exhibits several promising advantages over the conventional total knee replacement (TKR) (4).

Certainly, the choice between unilateral UKA and TKR depends on specific patient indications and preferences, with UKA offering a more conservative approach, quicker recovery, and potential advantages in isolated knee damage, while TKR provides a comprehensive solution for widespread knee arthritis, long-term durability, but is more invasive and may involve a longer recovery (5). Furthermore, UKA preserves a larger portion of the patient's natural knee joints, allowing for a more natural range of motion and more satisfactory long-term outcomes (6, 7).

Degenerative osteoarthritis often occurs simultaneously in both knees. According to Muraki et al. (8), 49.5% of people aged 60 years or older are given a diagnosis of bilateral knee degenerative osteoarthritis, and 20.4% of these individuals also experience symptoms of pain. Such patients must receive bilateral TKR.

Simultaneous TKR have been linked to enhanced patient satisfaction, cost-effectiveness, and expedited recovery, although the potential for contentious complications remains a subject of discussion (9–11).

A study reported that bilateral UKA may increase the risk of severe cardiac and pulmonary complications and lead to premature death at a rate similar to that of TKR (12). According to Biazzo et al. (13), bilateral UKA does not increase the risk of such complications and involves a shorter operation time and less postoperative blood loss.

In the meta-analysis conducted by Haowen Kwan et al. (14), it was demonstrated that bilateral UKA effectively reduces the length of operation and hospital stay. However, this study did not specifically address short-term postoperative outcomes, such as recovery and pain management. Considering that few studies have focused on the short-term postoperative recovery and cost-effectiveness of bilateral Oxford unicompartmental knee replacement, there remains uncertainty about whether bilateral UKA results in greater postoperative pain or poorer functional recovery compared to unilateral UKA. Additionally, the complications and costs associated with bilateral UKA have not been extensively explored.

This retrospective case-control study aims to compare bilateral UKA and unilateral UKA with respect to postoperative pain, costs, and complications. We hypothesize that undergoing bilateral UKA will not delay postoperative recovery or increase pain, while potentially reducing the average healthcare expenditure.

## Patients and method

### Population

This is a retrospective study. The data of patients who received Oxford knee replacement between July 2018 and June 2021 were included.

Patients were included if they (1) had a diagnosis of unicompartmental osteoarthritis (Kellgren-Lawrence grade 3–4) or spontaneous osteonecrosis of the medial compartment, and (2) had imaging that indicated the need for an Oxford knee replacement. Specifically, lateral view x-rays demonstrated a preserved posterior tibia, indicating a functionally intact anterior cruciate ligament (ACL), full-thickness lateral cartilage, a functionally normal medial collateral ligament, and an acceptable patellofemoral joint, meaning no lateral facet osteoarthritis with associated bone loss, grooving, or subluxation (15).

Patients were excluded if they received additional surgeries at the same time that they received Oxford knee replacement, with such surgeries including contralateral TKR or concurrent ACL reconstruction surgery. Patients who received unilateral Oxford UKA were assigned to Group A, and those who received bilateral Oxford UKA were assigned to Group B. The decision to choose a unilateral or bilateral UKA was influenced by the clinical conditions and choices of each patient, although surgeon's preferences also played a role.

### Data collection

Demographic information, that is, age, sex, body height, body weight, and body mass index, American Society of Anesthesiology score, preoperative Hb value, and operation time, was collected from the patients’ medical history. In addition to collecting data on patients’ range of knee motion before discharge, length of hospitalization, patient-controlled analgesia (PCA), total medical expenses encompassing hospitalization, medication, nursing care, anesthesia, and surgical costs, the study also documented any postoperative complications. Moreover, the minimal hemoglobin (Hb) levels and maximum visual analogue scale (VAS) scores within the initial 72 h following surgery were carefully recorded for analysis.

### Surgical protocol

Patients were given the option to choose between general and spinal anesthesia based on the personal preference of the surgeon or the recommendation related to anesthesia risks. After anesthesia was administered, the patient's limb was positioned by inflating a thigh tourniquet and placing the draped leg on a thigh support. The hip was flexed to approximately 30 degrees, allowing the leg to hang freely with the knee flexed at about 110 degrees. Care was taken to avoid placing the thigh support in the popliteal fossa to minimize the risk of damage to the popliteal vessels.

Before the operation, a minimally invasive midvastus or subvastus approach was adopted using inflatable or noninflatable tourniquets. During the operation, cruciate ligaments, lateral compartments, and patellofemoral joints were routinely examined to verify that the patients were eligible for Oxford UKA. A mobile-bearing UKA Oxford (Oxford Microplasty; Zimmer Biomet, Warsaw, IN, USA) was used for prosthesis. The surgeons used a drainage tube when necessary, with necessity determined on the basis of the blood loss during the operation and the preferences of the surgeons. All operations were conducted by senior orthopedic physicians.

### Postoperative rehabilitation and pain management

Postoperative patients receive NSAIDs and opioids. They choose between epidural or intravenous PCA before surgery, with an option to self-finance. All patients undergo hemoglobin testing. With the assistance of physical therapists, patients are encouraged to get out of bed and walk the day after the operation. Those who had persistent anemia continued to have their hemoglobin monitored. Homologous blood transfusions were performed when the hemoglobin concentration was under 8 g/dl or anemia symptoms were present. Postoperatively, the patients had an active range of motion and underwent partial weight-bearing on day 1 postoperation.

### Postoperative complications

Complications that occurred within 2 years after the operation and led to rehospitalization or reoperation were recorded. Major complications commonly associated with joint replacement surgeries include premature death, pulmonary embolism, proximal deep vein thrombosis (DVT), and myocardial infarction. Minor complications include superficial wound infection and distal DVT.

### Data analysis

Statistical analysis of the data was performed using SPSS statistical software. The chi-square test was performed to compare categorical variables (e.g., sex). The Mann–Whitney *U*-test and analysis of variance were used to compare the differences between the two groups.

Significance was indicated at *p* ≤ 0.05 with 95% confidence intervals.

## Result

A total of 232 patients who underwent Oxford knee replacement surgery between July 2018 and June 2021 were originally included. After four patients who underwent simultaneous contralateral TKA and two patients who underwent simultaneous ACL reconstruction were excluded, 226 patients were included for analysis. Among them, 170 underwent unilateral UKA (Group A) and 56 underwent bilateral UKA (Group B). Within Group A, none of the patients received contralateral knee joint replacement during the follow-up period. No statistically significant differences were observed in the basic patient characteristics of height, weight, and anesthesia risk assessment between the two groups (Table 1). However, the surgical time was significantly shorter in Group A than in Group B (110 min vs. 146 min, *p* < 0.05), and the decrease in Hb after surgery was greater in Group B than in Group A (1.4 g/dl vs. 1.2 g/dl, *p* < 0.05). No significant differences were observed in the length of hospitalization, VAS pain scores, or postoperative knee joint activity between the two groups. However, the total hospitalization cost was higher in Group B than in Group A (Table 2).

**Table 1:** Patient characteristics and demographics.

	Unilateral (*n* = 170)	Bilateral (*n* = 56)	*P* value
Age	68.0 (62.0, 73.0)	67.0 (63.3, 72.0)	0.858
Sex			0.826
Female	123 (72.4%)	39 (69.6%)	
Male	47 (27.6%)	17 (30.4%)	
Height	155.0 (150.0, 161.0)	156.0 (152.3, 161.0)	0.343
Weight	66.0 (59.8, 74.3)	67.0 (62.0, 76.0)	0.250
BMI	27.5 (25.3, 30.1)	27.8 (25.6, 30.0)	0.524
ASA score	2.0 (2.0, 2.0)	2.0 (2.0, 2.0)	0.725
Category			
OA	157 (92.4%)	55 (98.2%)	
SONK	13 (7.6%)	1 (1.8%)	
Anesthesia			
SA	48 (28.2%)	37 (66.1%)	
GE	122 (71.8%)	19 (33.9%)	

SA, spinal anesthesia; GE, general anesthesia; Chi-square test or Mann-Whitney *U*-test, median (IQR), was used for statistical analysis.

**Table 2:** Clinical outcomes.

	Unilateral (*n* = 170)	Bilateral (*n* = 56)	*P* value
VAS score	5.0 (3.0, 6.0)	5.0 (3.0, 7.0)	0.071
Hospital stay	6.0 (5.0, 6.0)	6.0 (5.0, 6.0)	0.679
Operation time(mins)	110.0 (59.0, 135.0)	146.0 (89.0, 174.75)	<0.001**
Range of motion(degree)	90.0 (90.0, 100.0)	90.0 (90.0, 102.5)	0.489
Hemoglobin difference(g/dl)	1.2 (0.8, 1.7)	1.4 (1.1, 1.9)	0.025*
Medical cost(NTD)	92,338.0 (89,413.5, 96,129.8)	149,929.0 (147,573.5, 155,683.5)	<0.001**
Complication	8	1	0.335

NTD, new Taiwan dollar; VAS, visual analogue scale. Mann-Whitney *U*-test, median (IQR), was used for statistical analysis. *P < 0.05, **P < 0.01.

Regarding the VAS pain scores (Table 3), the patients in Group A who received PCA had lower pain scores after surgery, and epidural PCA (EPCA) was more effective than intravenous PCA (IPCA). By contrast, no significant differences were noted in the pain scores between the patients with or without PCA after surgery in Group B.

**Table 3:** Pain scores based on anesthesia technique.

	EPCA (*n* = 76)		IPCA (*n* = 52)		NONE (*n* = 98)		*P* value
Group	*n*	mean ± SD	*n*	mean ± SD	*n*	mean ± SD	
Single (*n* = 170)	39	4.3 ± 1.94	42	4.5 ± 2.09	89	5.3 ± 2.31	0.034*
Bilateral (*n* = 56)	37	5.1 ± 2.38	10	5.6 ± 2.72	9	7.1 ± 2.42	0.104

EPCA, epidural PCA; IPCA, intravenous PCA; NONE, No PCA. Pain scores are presented as mean ± SD. ANOVA was used for statistical analysis. *P < 0.05.

Eight complications occurred within 2 years of follow-up, with seven occurring in Group A (three insert dislocations, three inserts loosening at the tibia, and one wound dehiscence) and one occurring in Group B (i.e., stitch abscess formation) (Table 2).

Our study revealed that while unilateral UKA resulted in shorter surgical times and less decrease in hemoglobin levels after surgery compared to bilateral UKA, the total hospitalization cost was higher for the latter. Additionally, although PCA was more effective in reducing pain in patients who underwent unilateral UKA, no significant differences in pain reduction were observed among patients who underwent bilateral UKA with or without PCA. Finally, complications were more frequent in patients who underwent unilateral UKA.

## Discussion

In recent years, with improvements in implant design and advancements in surgical techniques, both navigated and conventional UKA have shown favorable long-term survival rates (16). Clinically, physicians frequently perform UKA, particularly in older adults. Problems related to recovery rate, pain severity, medical costs, and hospital stays have been reported after bilateral UKA operations. Although studies have discovered that bilateral UKA does not increase the risk of complications, those comparing the postoperative pain and medical costs between surgeries are scant. Therefore, the current study compared the early postoperative functional recovery, complications, and medical costs between bilateral Oxford UKA and unilateral Oxford UKA.

The main hypotheses were verified in this study. The postoperative recovery and pain did not differ significantly between the patients who received unilateral UKA (Group A) and those who received bilateral UKA (Group B). Both reported VAS scores of 5 and could bend their knee joints up to 90° (*p* > 0.05). This is consistent with the findings of Clavé et al. (17), which indicated that patients receiving bilateral UKA do not use more pain medication or have a lower recovery rate than those receiving unilateral UKA. Although Clavé et al. had a smaller sample size, their results were similar to those of the present study. However, present study did not conduct long-term follow-ups, and therefore our results did not accurately reflect the patients’ postoperative functional recovery.

The current study analyzed postoperative pain. For Group A, pain was less severe when EPCA or IPCA was performed. Although EPCA was more effective than IPCA at alleviating pain, EPCA and IPCA did not differ significantly in their effectiveness for Group B, which may be due to the number of patients included in this study.

In this study, both patient groups had an average hospitalization duration of 6 days, with no significant differences observed between the groups. According to the meta-analysis by Haowen Kwan et al. (14), the length of hospital stay for unilateral UKA has been reported to range from 6.7 days to as short as 2.5 days, while for bilateral UKA, it varies from 14 days to 1.7 days. Recent studies have shown a trend toward shorter hospital stays, which may be attributed to advancements in pain management. For instance, Jensen et al. even reported an average hospital stay of just one day for UKA (18).

This variation in hospitalization duration is likely influenced by regional customs and differences in healthcare systems. In Taiwan, the comprehensive healthcare system typically necessitates a longer hospital stay, usually ranging from 5 to 7 days postoperatively. During this period, patients receive thorough care, including pain management, rehabilitation, and the removal of drainage tubes and Foley catheters.

In terms of healthcare expenditures, our study included costs related to hospitalization, nursing care, medication, surgery, and anesthesia. Notably, Group B incurred higher medical expenses (NT$149,929) compared to Group A (NT$92,338). Given that the length of hospital stay and post-operative care were similar between the two groups, it can be inferred that the additional expenses were primarily associated with surgical and anesthesia fees.

Nevertheless, Group B spent considerably less on average than Group A did in the long-term because they did not require rehospitalization or reoperation. This finding is consistent with those of other studies (13, 19).

Regarding complications, Group B lost more blood (1.4 g/dl) than Group A did (1.2 g/dl; *p* < 0.05), which is consistent with the findings by Biazzo et al. (13), who reported 3.13 g/dl of blood loss for patients receiving bilateral UKA and 2.47 g/dl for those receiving unilateral UKA. Both groups lost less blood than the patients who underwent TKR did. According to Fu et al. (20), bilateral TKR not only involves greater blood loss during operation but also increases the need for blood transfusion.

More patients from Group A reported having postoperative complications that led to rehospitalization than patients from Group B. Seven patients from Group A were given diagnoses of postoperative complications. Three of these patients received a diagnosis of insert dislocation, three received a diagnosis of insert loosening at the tibia, and one received a diagnosis of wound dehiscence. Only one patient from Group B had a postoperative complication, namely a wound infection.

Prosthesis infections, with an incidence ranging from 0.5% to 3%, result from the complex interaction of multiple factors, including bacterial contamination, the prosthesis itself, and host vulnerability (21). Additionally, complications such as deep vein thrombosis (DVT), cardiorespiratory issues, and premature death, which are typically associated with total knee replacement (TKR), were not reported in this study due to the lower complication rates observed with unicompartmental knee arthroplasty (UKA) compared to TKR.

Group A and Group B did not differ significantly with respect to postoperative complications; however, more patients from Group A had complications than did those from Group B. These results are consistent with the results of Malahias et al. (12), which revealed that bilateral UKA is safe and did not differ significantly from unilateral UKA in terms of postoperative complications. Nonetheless, bilateral UKA involves a higher likelihood of requiring postoperative blood transfusion.

This study has several limitations. First, as a retrospective study, its results may not be as reliable as those from a randomized controlled trial. Additionally, there is a discrepancy in the sample sizes between the two groups (170 vs. 56). This imbalance reflects the real-world distribution of patients who underwent single-stage bilateral and unilateral medial unicompartmental knee arthroplasty at our institution during the study period. However, this difference in sample sizes may have influenced the generalizability and robustness of our findings. Furthermore, due to the limited follow-up data available at the time of this study, we were unable to conduct a comprehensive survival analysis.

In addition, the patients who received bilateral UKA were relatively healthy, which may have led to selection bias because the patients experienced few postoperative complications. Nevertheless, the demographic characteristics between the patient groups did not significantly differ. Furthermore, because the case documentation method was adopted, the effects of cumulative doses of pain medication could not be thoroughly analyzed and only basic results, such as those regarding the effects of PCA use, were obtained. The patients’ physical functions could only be inferred by their knee mobility being restored, and no real record containing knee function scores was available. Because this study compared the postoperative hospitalization of patients, the results regarding joint mobility may be generalizable to all patients with knee arthroplasty, which counterbalances the shortcomings of this study.

Future studies are needed to validate our results and provide a more comprehensive analysis.

The findings regarding early postoperative functional recovery and pain did not significantly differ between the patients who received unilateral UKA and those who received bilateral UKA. Although bilateral UKA may incur greater blood loss and higher medical costs than unilateral UKA does, it does not lead to a need for rehospitalization and reoperation in the long-term and does not involve the risks associated with anesthesia. Although the current study has some limitations, this study provided a detailed analysis of the postoperative pain incurred by unilateral and bilateral UKA. The findings may assist clinicians in managing the pain of patients who receive UKA. Further research is required to verify these findings and clarify the long-term effects of UKA.

## Conclusions

Length of hospital stay, postoperative pain severity, and complications do not significantly differ between patients who receive bilateral Oxford UKA and those who receive unilateral Oxford UKA. Although bilateral Oxford UKA requires a longer surgery time and incurs higher medical expenses than unilateral Oxford UKA does, it does not involve many of the risks associated with anesthesia and involves lower overall health-care expenses. Therefore, if the indications permit, bilateral Oxford UKA may indeed be considered a safe option for patients, potentially reducing the anesthetic risks associated with staged surgery and alleviating the overall burden of hospitalization costs.

## Data Availability

The original contributions presented in the study are included in the article/Supplementary Material, further inquiries can be directed to the corresponding author.
